# Role of Outpatient Engagement and Medication Adherence in Managing Recurrent Suicidality Amidst Adverse Social Determinants: A Case Series

**DOI:** 10.7759/cureus.111439

**Published:** 2026-06-24

**Authors:** Oladele D Owasoyo, Joshua Van Le, Tinashe Muzorewa, Raheel Makhani, Obiora Onwuameze

**Affiliations:** 1 Psychiatry, Southern Illinois University School of Medicine, Springfield, USA

**Keywords:** major depressive disorder diagnosis and treatment, major trauma, out patient follow-up, self-harm, suicide behavior

## Abstract

Recurrent suicidality is a persistent clinical challenge characterized by frequent emergency department (ED) utilization and limited response to acute care models. We present a case series of four patients followed over three years with repeated suicidal ideation, multiple suicide attempts, and recurrent hospitalizations. Shared risk factors included major depressive disorder (MDD), borderline personality disorder (BPD), repetitive self-injury, medication overdose, family history of suicide, and social isolation. These vulnerabilities were compounded by trauma, polysubstance use involving cocaine, cannabis, and prescription or over-the-counter medications, as well as adverse social determinants of health, particularly housing instability and unemployment. Poor outpatient engagement and medication non-adherence were linked to increased suicide attempts, ED visits, and rapid readmissions. In contrast, improved outpatient continuity, treatment adherence, and structured support were linked to reduced suicidality and healthcare utilization. These findings underscore the importance of longitudinal, multidisciplinary care in managing recurrent suicide risk.

## Introduction

Recurrent suicidality encompassing both suicidal ideation and behavior represents a critical challenge characterized by frequent healthcare utilization that often defies traditional, acute risk assessments [1]. While single-encounter frameworks focus on immediate crises, they frequently overlook the longitudinal complexity of patients facing a compounding risk of severe personality pathology, chronic trauma, and social instability [2].

This retrospective case series examined four patients with recurrent suicidality identified over a three-year period within a large healthcare system. Using a purposive sampling strategy, patients were selected from an electronic health record (EHR)-derived database based on recurrent suicidality presentations, including repeated emergency department (ED) visits and psychiatric hospitalizations for suicidal ideation or attempts. Clinical data were obtained through structured chart review and included psychiatric diagnoses, self-harm behaviors, healthcare utilization, psychosocial stressors, family history, and treatment factors such as outpatient engagement and medication adherence. All data were de-identified. The study was approved by the Institutional Review Board of Southern Illinois University, Springfield, IL, USA, which granted a waiver of informed consent due to the retrospective design and minimal risk to participants (approval number: 25-767).

This case series examines how diverse etiologies ranging from intimate partner violence to the progression of self-injury converge into persistent patterns of suicidality and, hence, numerous psychiatric readmissions [3,4]. The objective is to examine these intersecting vulnerabilities and highlight how outpatient engagement, medication adherence, and social determinants of health influence the trajectory of recurrent suicidal behavior beyond acute stabilization.

## Case presentation

Patient 1

The patient was a 41-year-old woman at the beginning of the observation period who presented repeatedly to the ED for suicidality over a three-year period. Her primary psychiatric diagnosis across encounters was recurrent major depressive disorder (MDD), severe, without psychotic features. Comorbid psychiatric conditions included alcohol use disorder, polysubstance use disorder (cocaine, benzodiazepines, phencyclidine, and cough/cold preparations), borderline personality disorder (BPD), and anxiety. Medical comorbidities included hypertension, diabetes mellitus type 2, seizure disorder, hyperlipidemia, and sleep apnea.

Between 2018 and 2021, the patient presented to the ED on 14 occasions with suicidality-related chief complaints. Psychiatry consult was documented consistently at each encounter, and risk assessments frequently characterized the patient as being at moderate to high risk, particularly in the context of acute psychosocial stressors and substance use. Suicide risk remained persistently elevated throughout the observation period as reflected by the Columbia-Suicide Severity Rating Scale (C-SSRS) and Suicide Assessment Five-step Evaluation and Triage (SAFE-T) protocol assessments [5,6].

Across the 14 encounters, six visits involved documented suicide attempts, all involving cutting or lacerations to the wrists or forearms. These impulsive acts typically occurred shortly before ED arrival during periods of acute emotional distress under the influence of alcohol and prescription drugs. Prior to the study period, the patient had a history of prescription drug abuse, including legal trouble for writing fake prescriptions. In the remaining eight encounters, the patient endorsed active suicidal ideation without a suicide attempt. These visits frequently included explicit suicidal plans, most commonly plans to cut herself, though no additional suicide methods were consistently documented. The patient had several visits in which her suicidal thoughts resolved after she detoxified from alcohol and other substances.

Blood alcohol concentrations were documented between 0.211-0.352 g/dL for most ED visits, which confirmed alcohol intoxication [7]. Apart from alcohol use, there was an accompanying drug overdose on two occasions, which involved drugs such as fluoxetine, cyclobenzaprine, lorazepam, clonazepam, and Coricidin HBP. The patient also had an extensive smoking history of 1/4 to 1/2 pack per year during the study period, beginning in her teenage years. The patient’s family history was significant for alcohol use disorder in her father and brother, as well as unspecified psychiatric illnesses on her mother’s side, with her maternal uncle having committed suicide.

Documented psychosocial stressors included interpersonal conflict, joblessness, relationship instability, financial strain, unstable housing, and difficulty maintaining outpatient care. The patient reported a history of both physical and emotional abuse, which was referenced in several encounters as contributing to longstanding depressive symptoms and chronic suicidality. This included her first husband and several partners thereafter. Protective factors included family relationships and job-oriented goals.

Her care was very inconsistent, with long periods of no-show appointments. She also maintained short periods of sobriety (two to four months). She found success when seeing fewer practitioners with more frequent follow-up appointments, but due to medical comorbidities, referral was unavoidable. Treatment during ED encounters involved psychotropic medication changes, including antidepressant therapies, anticonvulsants, and sleep aids. Each encounter involved a multidisciplinary approach with social workers and substance abuse counselors on board to help the patient acquire housing and reduce her likelihood of relapse. Disposition varied, with some visits resulting in inpatient psychiatric admission or medical admission with psychiatric follow-up. Several visits resulted in discharge for outpatient follow-up as well. These patterns reflected intermittent engagement with outpatient care and substance use treatment across the observation period, during which periods of sobriety were associated with reduced suicidality and relapse with recurrence of symptoms.

In recent years, the patient has significantly reduced ED utilization for suicidality. She attributes this to remission of her alcohol use disorder after seeing her daughter’s struggle with substance abuse. She obtained disability income and stable housing with the local shelter housing assistance program. Although she still struggles with mental illness, her suicidal thoughts and self-harm behaviors have reduced with a tailored medication regimen including duloxetine, gabapentin, hydroxyzine, quetiapine, and trazodone.

Patient 2

The patient was a 21-year-old female at the beginning of the observation period with a long-standing psychiatric history of MDD with psychotic features. Her clinical profile was further complicated by comorbid post-traumatic stress disorder (PTSD), BPD, and generalized anxiety. Her medical history also included asthma, migraines, panic disorder, and chronic vitamin D deficiency.

Between 2020 and 2023, the patient presented to the ED on 34 occasions for suicidality, 14 of which involved active suicide attempts. Notably, during this period from 2020 to 2021, the patient experienced housing instability and had 13 psychiatric hospitalizations, with six documented suicide attempts. Clinical assessments using the C-SSRS and SAFE-T protocol frequently indicated a moderate-to-high suicide risk [5,6]. During assessments, the patient also reported chronic pain and recurrent medication non-adherence before admission. The methods used in her 14 attempts were of high lethality and varied widely. They include one attempt by hanging, one with a combination of cutting and choking, one cutting and overdosing, two self-cutting, one attempt at drowning in a lake, one swallowing of two razor blades, one positioning herself on a railroad track, and seven overdosing on her medications, including Flexeril, Midol, Prozac, Depakote, naproxen, and hydroxyzine at different times. The patient consistently presented with command auditory hallucinations instructing her toward self-harm and reinforcing a sense of worthlessness.

According to the patient’s report and assessment during all the admissions, the patient’s suicidality is rooted in a sexual assault at age 14, which led to a pregnancy and subsequent therapeutic abortion. Following the assault, she was placed in foster care and experienced multiple adolescent hospitalizations until age 18. These events serve as primary, recurrent triggers for her depressive crises, as most of the patient’s admissions fell on either Mother’s Day or the anniversary of her abortion/sexual assault. Other psychosocial stressors reported include chronic interpersonal conflict with her mother and her mother’s partner, whom she describes as unsupportive. The patient’s family history was significant for psychiatric pathology, as her mother had a diagnosis of bipolar disorder and PTSD, with a history of three suicide attempts.

It is noteworthy that the patient was managed by multiple clinical departments, especially the admission following her presentation for foreign body ingestion. Computed tomography of the abdomen and pelvis revealed two radiopaque objects consistent with the razor blades reported by the patient. Esophagogastroduodenoscopy (EGD) was performed to retrieve one object; however, the second object was managed conservatively to minimize the risk of visceral perforation.

By 2022, the patient reported a significant reduction in ED utilization, which she attributed to improved engagement with a community-based outpatient support. She demonstrated improved use of coping strategies, including cleaning. The patient identified frequent transitions in her care team, a history of sexual trauma, and a recent ectopic pregnancy in 2021 as chronic triggers for her suicidality. At that time, she was managed using a multidisciplinary approach that integrated improved medication adherence with a refined pharmacologic regimen, including antipsychotic therapy, antidepressant, and anxiolytic agents. She further reported expansion of her outpatient care resources, including the addition of a community support worker, with consideration for incorporation of a health coach through a structured community-based program to facilitate long-term stabilization and recovery.

Patient 3

The patient was a 50-year-old woman at the beginning of the observation period who had an extensive psychiatric history, including MDD, recurrent and severe with psychotic features, generalized anxiety disorder, BPD, and polysubstance use disorder involving cocaine and cannabis. Her medical history was significant for chronic pain and traumatic brain injury secondary to a motorcycle accident in her 30s, as well as chronic obstructive pulmonary disease (COPD), hypothyroidism, hypertension, and hyperlipidemia.

Between 2020 and 2023, she presented to the ED on 15 occasions for suicidality, each involving suicidal ideation with intent and plan. She reported four prior suicide attempts by medication overdose, one resulting in a seven-day coma. Across encounters, SAFE-T protocol documentation consistently reflected high suicide risk [6]. Notably, she rarely endorsed a single suicidal plan, instead describing multiple concurrent methods, including overdose, walking into traffic, cutting, or stabbing herself.

A consistent pattern preceding each hospitalization was active cocaine and cannabis use in the days before presentation. During several admissions, she also endorsed command auditory hallucinations instructing her to kill herself. Although manic-like symptoms and psychosis raised concern for bipolar I disorder, this diagnosis was ultimately ruled out due to the temporal association with stimulant use and the absence of manic symptoms during prolonged periods of sobriety.

The patient’s psychosocial history was marked by unstable housing, alternating between staying with friends, her adult son, or homeless shelters. The patient had unstable housing during more than 60% of her ED visits. She reported a family history of suicide attempt by her sister, a trauma history of childhood sexual abuse perpetrated by her brother, and multiple episodes of domestic violence in adulthood. Socially, she was largely isolated and estranged from two of her three adult children, maintaining intermittent contact with one son. She had completed 11th grade and was unemployed while receiving Supplemental Security Income (SSI).

Throughout admissions, she consistently presented with depressed mood, hopelessness, and suicidal ideation. Her medical comorbidities frequently contributed to her psychiatric distress; she reported that chronic pain and dyspnea related to COPD significantly diminished her quality of life and served as recurrent triggers for suicidal thoughts. At other times, suicidal ideation was driven by shame and frustration related to relapse on cocaine. No acute medical or neurological etiology was identified to account for her psychiatric decompensations.

During hospitalizations, the patient received pharmacologic management targeting mood symptoms, psychosis, and anxiety, with transient improvement in suicidal ideation. However, her clinical course was characterized by poor adherence to prescribed medications and limited engagement with outpatient psychiatric follow-up following discharge. These factors frequently resulted in rapid readmissions, often occurring within two to four weeks.

Substance use remained a major perpetuating factor. Despite repeated counseling and multiple offers for inpatient substance use rehabilitation, she consistently declined formal treatment. Given the high frequency of hospitalizations and persistent difficulty adhering to outpatient care, transfer to an extended care facility was recommended. At the time, the patient declined this option, expressing a preference to live with family and asserting that she would be able to maintain stability in a less restrictive setting.

After the last psychiatric admission in 2023, the patient was discharged to an extended care facility (ECF). The structured environment in this facility ensured medication adherence and consistent outpatient follow-up, which resulted in clinical stability. Thereafter, she did not present to the ED until shortly after she was transitioned from the ECF to her own apartment. At this point, she had a medication lapse, decompensation, and readmission to the ED in November 2025.

Patient 4

The patient was a 19-year-old woman at the beginning of the observation period with a psychiatric history of recurrent MDD, BPD, PTSD, and generalized anxiety disorder. Relevant medical comorbidities included asthma, hypothyroidism, obesity, and episodes described as psychogenic non-epileptic seizures. The observation period spanned from 2019 to 2022. Structured suicide risk assessments using the SAFE-T protocol and the C-SSRS frequently indicated moderate to high risk [5,6].

Across the documented period, the patient presented to the ED on 37 occasions for suicidal ideation or suicide-related behaviors, often in close temporal clusters with next-day returns and multiple visits within several days of discharge. Suicide attempts were documented in 16 encounters. Self-poisoning via prescribed and over-the-counter medications, particularly acetaminophen, was the most frequent method and accounted for multiple medically serious overdoses requiring inpatient medical management. Additional methods included toxic ingestion of detergent, cutting with self-inflicted lacerations, attempted drowning, and impulsive acts such as running into traffic. The patient also engaged in near-lethal, high-intent behaviors, including an interrupted hanging attempt. In the remaining encounters, she endorsed active suicidal ideation with intent and plan without a completed attempt. Despite repeated overdoses requiring inpatient medical management, the patient did not achieve sustained psychiatric stabilization.

Psychiatric evaluations consistently characterized her suicidality as chronic and baseline rather than episodic. Encounters documented emotional dysregulation, interpersonal sensitivity, impulsivity, and fear of abandonment, consistent with prominent personality-related vulnerability. Repetitive cutting behaviors were noted across encounters, reflecting a pattern of recurrent self-injury. Distressing perceptual experiences were intermittently reported and conceptualized as trauma-related rather than primary psychotic phenomena.

Psychosocial adversity was prominent as the patient experienced the death of a parent during the observation period, which coincided with increased emergency utilization. She reported acute distress after witnessing the suicide of a cousin via video call. This was repeatedly cited as a precipitant for the patient’s crises. Interpersonal conflict, perceived abandonment, and unemployment were recurrent stressors. ED care consistently included medical screening, psychiatric consultation, and safety planning.

Key clinical characteristics, psychotropic medications, and treatment approaches across the four cases are summarized in Table 1.

**Table 1:** Comparison of suicidality-related emergency department (ED) visits, suicide attempt frequency, major contributing factors, selected psychotropic medications, and treatment approaches across the four cases Suicidality-related ED visits and suicide attempt counts reflect events documented during each patient's observation period. Psychotropic medications and treatment approaches represent selected interventions documented during each patient's observation period and are not intended to reflect all medications, therapies, services, or treatments received.

Patient	Age at the Start of the Observation Period	Suicidality-Related ED Visits	Suicide Attempts	Major Contributing Factors	Psychotropic Medications	Treatment Approaches
1	41 years	14	6	Major depressive disorder, borderline personality disorder, alcohol use disorder, anxiety symptoms, chronic suicidality, family history of suicide, recurrent alcohol relapse, housing instability, and psychosocial stressors	Duloxetine, fluoxetine, trazodone, clonidine, hydroxyzine, divalproex sodium, gabapentin, quetiapine, disulfiram, naltrexone, venlafaxine	Repeated psychiatric hospitalization, crisis stabilization, suicide precautions, psychotropic medication management and optimization, alcohol withdrawal management, substance use counseling, inpatient rehabilitation referral, outpatient psychiatry, outpatient counseling/psychotherapy, psychoeducation, safety planning, and community recovery support services
2	21 years	34	14	Major depressive disorder, borderline personality disorder, post-traumatic stress disorder (PTSD) related to prior sexual trauma, chronic suicidality and self-injurious behavior, intermittent psychotic symptoms, medication nonadherence, family history of suicide, housing instability, interpersonal conflict, and pregnancy loss	Fluoxetine, paroxetine, sertraline, trazodone, hydroxyzine, prazosin, divalproex sodium, lamotrigine, lithium, oxcarbazepine, aripiprazole, haloperidol, paliperidone, quetiapine, risperidone, ziprasidone	Repeated psychiatric hospitalization, crisis stabilization, suicide precautions, psychotropic medication management, psychotherapy, partial hospitalization, outpatient psychiatry, outpatient psychotherapy, family psychoeducation, coping-skills training, safety planning, and community support services
3	50 years	15	0	Major depressive disorder, borderline personality disorder, PTSD, generalized anxiety disorder, cocaine use disorder, cannabis use disorder, chronic suicidality, chronic auditory hallucinations, family history of suicide, chronic pain, psychosocial stressors, grief/loss, and housing instability	Duloxetine, venlafaxine, clonazepam, hydroxyzine, prazosin, divalproex sodium, gabapentin, haloperidol, olanzapine, quetiapine, risperidone	Repeated psychiatric hospitalization, crisis stabilization, suicide precautions, psychotropic medication management and optimization, psychotherapy, psychoeducation, substance abuse counseling, milieu therapy, recreational therapy, outpatient psychiatry, outpatient psychotherapy, intensive outpatient psychotherapy referral, case management services, safety planning, and community mental health follow-up
4	19 years	37	16	Major depressive disorder, borderline personality disorder, PTSD, emotional dysregulation, chronic suicidality, family history of suicide, interpersonal conflict, perceived abandonment, death of a parent, witnessing a relative's suicide, unemployment, and psychosocial stressors	Citalopram, paroxetine, sertraline, trazodone, venlafaxine, hydroxyzine, prazosin, lamotrigine, lithium, oxcarbazepine, topiramate, paliperidone palmitate, quetiapine	Repeated psychiatric hospitalization, crisis stabilization, suicide precautions, psychotropic medication management and optimization, electroconvulsive therapy, outpatient psychiatry, outpatient psychotherapy, safety planning, and community mental health follow-up

## Discussion

This case series highlights the complex interplay between severe personality pathology, family history, chronic trauma, and high-frequency healthcare utilization. Outcomes were strongly influenced by outpatient engagement and medication adherence, with poor follow-up linked to instability and frequent healthcare use, and improved continuity associated with reduced suicidality.

The multiple admissions of Patient 1 underscore the role of intimate partner abuse as a primary driver of suicidality, a correlation well-supported by research linking adult victimization to recurrent self-harm [3]. However, a critical finding in Patient 2 is the presence of "anniversary reactions" as primary triggers for suicidal crises. Most of the patient’s admissions consistently aligned with Mother’s Day and the anniversaries of her sexual assault and subsequent abortion. Recent literature highlights that trauma anniversaries can lead to a significant resurgence of PTSD symptoms, including command hallucinations and suicidality [8]. Our patient’s case supports these findings, demonstrating how specific temporal cues can reactivate deep-seated trauma, leading to life-threatening self-harm. The patient’s history of deliberate foreign body ingestion (DFBI) represents one of the most resource-intensive and clinically fraught aspects of her care, coupled with her BPD. Recent multicenter studies indicate that DFBI is disproportionately prevalent in patients with BPD (63%) and is frequently associated with suicidal intent [9]. Patient 3’s case illustrates how recurrent suicidality may be sustained by the interaction of illicit substance use and traumatic brain injury. Use of illegal substances, including cocaine and cannabis, is associated with increased suicide risk through acute disinhibition, mood instability, and impulsivity [10]. Traumatic brain injury further increases vulnerability through cognitive and emotional dysregulation, which may limit coping capacity and reduce responsiveness to standard crisis interventions [11].

The cases of Patients 2 and 3 highlight how early-life trauma, chronic pain, and difficulty sustaining treatment engagement may contribute to ongoing suicide risk. Childhood abuse is linked to long-term psychiatric vulnerability and elevated rates of suicidal behavior [12]. While chronic pain independently increases suicide risk, particularly in the setting of psychiatric comorbidity, poor adherence to treatment may further exacerbate risk by limiting symptom stabilization and continuity of care [13,14]. The convergence of these factors illustrates how developmental, somatic, and care-related vulnerabilities may interact to maintain recurrent suicidality.

Self-poisoning via prescribed and over-the-counter medications was common among Patients 1, 2, and 4. This led to frequent suicide-related admissions without achieving long-term psychiatric stability. This repetitive pattern underscores the role of medication overdose as a predominant method in suicide attempts [15]. The recurrent suicidal admissions for Patients 1, 2, and 3 highlight the compounding risk of social isolation and a positive family history. These factors likely created a state of chronic vulnerability, echoing research that identifies inadequate social support as a primary factor distinguishing individuals with multiple suicide attempts from those with a single episode [16]. The clinical history of repetitive cutting in all our patients underscores the significant risk of transition from non-suicidal self-injury (NSSI) to suicidal behavior. This observation is consistent with recent meta-analytic data identifying NSSI as one of the strongest prospective predictors of suicide attempts, often surpassing the predictive power of a history of suicidal intent alone [4].

All four patients had MDD and BPD. MDD and BPD are common drivers of psychiatric crisis, which can lead to elongated recovery time and high levels of suicidality [17]. MDD is highly prevalent in emergency department populations, with approximately 26.5% of patients screening positive for MDD and demonstrating increased subsequent healthcare utilization [18]. Personality disorders, including BPD, also account for a substantial proportion of acute psychiatric presentations, representing approximately 26% of emergency mental health presentations and 25% of inpatient admissions [19]. All four patients also endorsed a family history of suicide. Notably, Patient 4 reported acute distress after witnessing a relative’s death by suicide via a real-time video call. This is consistent with the literature on familial transmission; this study identifies family history as a robust predictor of high-frequency suicidality [16,20]. This relationship underscores the necessity of intensive clinical monitoring for patients with a familial background of suicide, as they exhibit a markedly higher propensity for recurrent, life-threatening behaviors.

Furthermore, in all four patients, clinical instability was heavily influenced by housing instability, which is one of the social determinants of health. It has been reported that housing instability can be positively associated with suicide ideation [2]. Joblessness functioned not only as an acute trigger for most episodes in all four patients but also as a persistent barrier to long-term recovery. The recurrent nature of the suicidal admissions in these four patients underscores the compounding impact of unemployment on mental health stability. Such patterns reflect the findings that while a history of prior attempts is a primary predictor of future risk, socio-economic factors like unemployment significantly increase the likelihood of repetitive suicidal behavior [1]. Beyond socio-economic stressors, clinical instability was evidenced by the patients' collective difficulty in maintaining consistent outpatient follow-up. This pattern of disengagement has been associated with increased suicide risk [14]. Notably, in three of the four patients, consistent outpatient engagement appeared to serve as a protective factor, markedly reducing suicidal behavior during and following the study period. This supports current evidence suggesting that outpatient care could be essential for stabilizing high-risk individuals.

Limitations

This study has some notable limitations. First, the sample size is small and limited to four patients, which restricts the generalizability of the findings. Second, all cases were derived from a single clinical center, which may limit applicability to other healthcare systems or populations. Additionally, the retrospective nature of the case series review relies on documentation across multiple electronic health records and may not capture all psychosocial variables influencing suicidality. The observation period overlapped with the COVID-19 pandemic, although available clinical documentation did not identify pandemic-related factors as major contributors to suicidality in these cases. Despite these limitations, the detailed longitudinal observation of these patients provides insight into patterns of recurrent suicidality and highlights the need for sustained, trauma-informed outpatient care.

## Conclusions

This case series highlights that while triggers for recurrent suicidality are etiologically diverse, they converge into a singular, self-perpetuating cycle of clinical and social instability. By conceptualizing recurrent suicidality as a longitudinal phenomenon rather than a series of isolated events, we demonstrate that episodic, crisis-driven admissions are insufficient for patients facing a constellation of trauma, family history, traumatic brain injury, and housing instability. These findings urge the medical community to move beyond single-encounter frameworks, recognizing that high-frequency healthcare utilization is a signal of persistent, cumulative risk. Ultimately, this study serves as a call for a proactive, tailored pharmacological regimen and trauma-informed outpatient engagement that addresses medication non-adherence and socio-economic vulnerabilities at the root of chronic suicidality. Future research is needed to explore and establish the relationship between drug overdose and patterns of recurrent suicidal behavior in patients with complex psychiatric comorbidities.
